# Nanoencapsulation Strategies for Stabilization and Delivery of Beetroot (*Beta vulgaris*) Bioactive Compounds: Current Advances and Future Perspectives

**DOI:** 10.3390/ijms27219986

**Published:** 2026-11-07

**Authors:** Oscar Zannou, Mansuri M. Tosif, Rıdvan Arslan, Reza Tahergorabi, Gulden Goksen

**Affiliations:** 1 Department of Food Technology, Vocational School of Technical Sciences at Mersin Tarsus Organized Industrial Zone, Tarsus University, Mersin 33100, Türkiye; tosifmansuri444@gmail.com (M.M.T.); rdarslan63@gmail.com (R.A.); 2 Food and Nutritional Sciences Program, North Carolina A&T State University, Greensboro, NC 27411, USA; rtahergo@ncat.edu

**Keywords:** nanoencapsulation, beetroot bioactives, nanocarriers, controlled release, functional food systems

## Abstract

Beetroot (*Beta vulgaris*) is a rich source of bioactive compounds, including betalains, phenolic compounds, and dietary nitrates, which are associated with strong antioxidant, anti-inflammatory, and cardioprotective properties. The practical application of these compounds in food and nutraceutical systems is significantly limited by their high sensitivity to environmental conditions such as pH, temperature, light, and oxygen, leading to rapid degradation, reduced stability, and low bioavailability. Nanoencapsulation has emerged as a promising strategy to overcome these challenges. Nanoliposomes, polymer-coated liposomes, chitosan-based nanogels, and nanoemulsions have emerged as promising nanocarrier platforms for improving the physicochemical stability and gastrointestinal fate of beetroot bioactive compounds. Nanoliposomes offer advantages in terms of nanoscale particle dimensions and relatively high loading capacities, whereas polymeric coatings can provide an additional protective barrier against premature release during gastrointestinal transit. Chitosan nanogels are particularly attractive because of their mucoadhesive properties and potential to promote prolonged and controlled release, while double nanoemulsions provide efficient dispersion of beetroot bioactive within nanosized droplets and can improve colloidal stability. Despite significant progress, challenges related to large-scale production, cost-effectiveness, regulatory approval, and long-term safety remain. Future research should focus on the development of sustainable, biopolymer-based nanocarriers, integration with green extraction technologies, and advanced delivery systems with improved targeting and responsiveness. Overall, nanoencapsulation represents a transformative approach to unlocking the full potential of beetroot bioactive compounds for industrial and health-related applications.

## 1. Introduction

Food bioactives are non-essential, non-nutrient molecules naturally present in small amounts in foods (e.g., polyphenols, carotenoids, phytosterols, glucosinolates, organosulfur compounds) that exert biological effects in the body beyond basic nutrition [1,2]. Higher intake of plant bioactives is linked to lower incidence of cardiovascular disease, cancers, diabetes, obesity, and neurodegenerative disorders [1,2,3,4]. There is growing development of functional foods and nutraceuticals enriched with bioactives to support the prevention or management of Non-communicable diseases (NCDs) [1,3]. Encapsulation and nano-delivery systems are being used to improve the often-low bioavailability of these compounds [5,6].

Beetroot (*Beta vulgaris* L.) is widely recognized as a functional food due to its dense content of nutrients and phytochemicals with physiological activity [7,8]. Beetroot provides a broad spectrum of bioactives, inorganic nitrate (NO_3_^−^), betalains, and phenolic compounds. Beetroot and leafy greens account for about 50–85% of dietary nitrate intake [9]. Dietary nitrate is almost completely absorbed and converted via the nitrate–nitrite–NO pathway to nitric oxide, supporting vasodilation, blood pressure regulation, and endothelial function [9,10,11]. Beetroot peel and stems are particularly rich in betalains, including betacyanins and betaxanthins, which have shown strong antioxidant, anti-inflammatory, chemopreventive, and potential cardiometabolic benefits in vitro and in vivo [8,10,12]. The phenolic compounds of beetroot include phenolic acids (chlorogenic, caffeic, ferulic, *p*-coumaric) and flavonoids (rutin, epicatechin, isoquercitrin, quercetin, etc.) with established antioxidant activity [10,13,14]. Other nutrients and phytochemicals determined in beetroot include vitamins (A, C, B-complex, folates), minerals (K, Na, Ca, Mg, Fe, Zn, Mn), fiber, carotenoids, saponins, glycine betaine, and organic acids (e.g., quinic acid), which contribute to antioxidant and metabolic effects [7,13,14].

Betalains are the primary bioactive compounds in beetroot. They are highly sensitive molecules, which makes them prone to degradation through (1) pH sensitivity, as they are stable only in a narrow pH range (4–6), (2) temperature (heat), as thermal processing (pasteurization, drying) accelerates their dehydrogenation and decarboxylation, leading to loss of color and antioxidant activity, (3) light exposure, as UV and visible light trigger photo-oxidation, (4) oxygen, as oxidative degradation leads to pigment bleaching, and (5) enzymes, as peroxidase and polyphenol oxidase can degrade these pigments. These limitations lead to the application of nanoencapsulation, which is essential to create a protective barrier against oxygen, light, and heat [15]. Moreover, it improves chemical stability, enables controlled release, and preserves color and bioactivity [12,15,16].

Our previously published review dealt with betalain extraction and encapsulation from different sources [15]. However, the present study focused on nanoencapsulation strategies for enhancing the stability, bioavailability, and functional performance of bioactive compounds derived from beetroot (*B. vulgaris*). This review aims to provide a comprehensive overview of nanoencapsulation strategies for enhancing the stability, bioavailability, and functional performance of bioactive compounds derived from beetroot (*Beta vulgaris*). It critically examines the physicochemical properties and degradation challenges of key compounds such as betalains, phenolics, and nitrates, followed by an in-depth analysis of various nanocarrier systems, including lipid-based, polymeric, and nanofiber-based delivery platforms. The review further explores the impact of nanoencapsulation on encapsulation efficiency, stability, and release behavior, as well as its applications in functional foods, nutraceuticals, and bioactive packaging. Additionally, sustainability aspects, including green extraction techniques and waste valorization, are discussed. Finally, current challenges, research gaps, and future perspectives are highlighted to guide the development of innovative and scalable delivery systems.

## 2. Brief Insight into Beetroot Bioactive Compounds and Functional Properties

**Figure 1:**
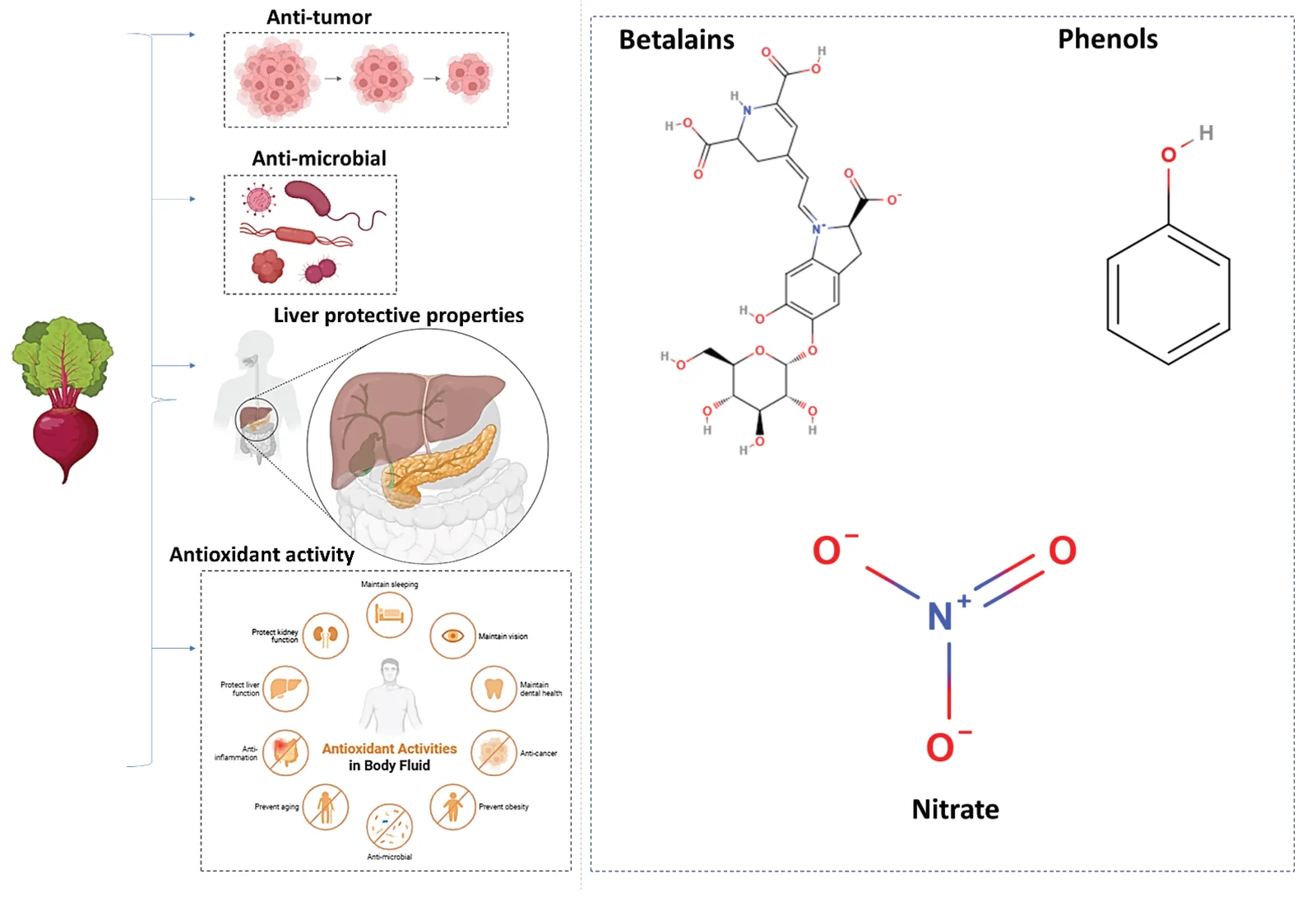
Major bioactive compounds and functional properties of beetroot.

Beetroot is a nutrient-dense root rich in distinctive phytochemicals that give it intense color and notable health effects. Core bioactives include betalain pigments, inorganic nitrate, and diverse phenolics, supported by vitamins, minerals, fiber, and amino acids (Figure 1). These underpin antioxidant, anti-inflammatory, and cardiometabolic benefits and make beetroot important in both nutrition and food technology (Table 1).

### 2.1. Betalains

Betalains are among the most characteristic phytochemical groups in red beetroot and are primarily responsible for its intense red-violet coloration. These water-soluble, nitrogen-containing pigments are divided into two structural classes, including betacyanins, which provide red-to-violet coloration, and betaxanthins, which contribute yellow-to-orange hues [12]. In red beetroot, betacyanins generally predominate and are mainly represented by betanin and its stereoisomer, isobetanin, whereas vulgaxanthin I and other amino acid-derived betaxanthins constitute an important fraction of the yellow pigments [17,18]. Betalains were found to be about 0.8–1.3 g/L in fresh juice, being typically 60% betacyanins (e.g., betanin) and 40% betaxanthins [9,13,19,20]. The average betalain content was found to be about 120 mg/100 g fresh weight, with the highest value determined in the peel [9]. Beetroot powders obtained from different production systems contained about 44.2–168.4 mg/100 g total betalains, illustrating that agricultural practices and processing history can substantially influence the final pigment concentration [21]. The HPLC–MS^n^ analysis of beetroot peel, flesh, and petiole showed that yellow beetroot cultivars could contain abundant betaxanthins while lacking detectable betacyanins, emphasizing that pigment composition is strongly genotype-dependent [18]. The same study reported that betanin/isobetanin pigments comprised a major portion of the relative peak area measured in red beetroot peel, whereas isobetanin relative peak areas were also high in leaf petioles (68.94% to 74.16%) of red colored cultivars. The authors determined that betacyanins were completely absent from the extracts of all analyzed parts of yellow beetroot [18]. Similarly, comparative analysis of 13 red beetroot varieties identified 30 betalains, comprising 18 betacyanins and 12 betaxanthins, and confirmed significant differences in total betalain content according to both genotype and root part [22]. These authors determined that betalain contents were 10.26–17.15 mg/g dm and 7.20–17.24 mg/g dm in varieties and root parts, respectively, differing significantly across varieties and root parts [22]. These observations indicate that reporting only “total betalains” may mask important differences in the qualitative pigment profile.

### 2.2. Phenolic Compounds

Phenolic compounds constitute an important fraction of the non-pigment phytochemicals of beetroot and contribute substantially to its antioxidant and other bioactivities. The total phenolic content in beetroot was reported to be in the range of 200–600 mg GAE/100 g DW [8,19,23,24]. Wruss et al. determined that the mean total phenolic content in the fresh juice prepared from 7 beetroot varieties ranged from 90 to 130 mg/100 g, with hydroxycinnamic acids, which represented the most abundant non-betalain group and accounted for up to 2.6% of total phenolics [25]. In a study, the organically and conventionally cultured beetroots were investigated for their content in bioactive compounds [26]. It was found that the total phenolic content varied from 564.4 to 1165.9 mg/100 g for the conventionally cultured beetroot, while from 914.5 to 1909.4 mg/100 g for the organically cultured beetroot. In the same study, the phenolic acids gallic acid, chlorogenic acid, caffeic acid, and *p*-coumaric acid, and the flavonoids quercetin-3-*O*-rutinoside, kaempferol-3-*O*-glucoside, and luteolin were identified in beetroot samples [26]. Another study investigated the phenolic content in beetroot juice, chips, powder, and cooked beetroot and determined the total phenolic content to be 367 mg/100 g, 75 mg/100 g, 51 GAE mg/100 g, and 279 mg/100 g, respectively [27]. Recent characterization of *B. vulgaris* roots identified compounds such as caffeic acid hexosides, sinapic acid rhamnosides, feruloyl-sinapic acid derivatives, and ferulic acid dihexoside, demonstrating that a considerable proportion of beetroot phenolics occurs in bound or conjugated forms rather than exclusively as free phenolic acids [28]. Overall, beetroot should be considered a complex source of phenolic compounds rather than simply a betalain-rich vegetable. Its phenolic content is controlled by genotype, tissue distribution, maturation, environmental conditions, extraction strategy, and processing history.

### 2.3. Other Bioactive Compounds

Nitrate (NO_3_^−^) is one of the key compounds in beetroot and beetroot by-products. Unlike betalains and phenolics, nitrate is not primarily associated with antioxidant capacity; its importance derives from its participation in the nitrate–nitrite–nitric oxide pathway [29]. Dietary nitrate can be reduced to nitrite and subsequently contribute to nitric oxide formation, providing a biochemical basis for the extensively investigated vascular effects of nitrate-rich beetroot products [9]. Nitrate was found in high concentrations in roots, stems, and leaves of beetroot, and the amount in the stems can reach up to 359 mg/kg, displaying a higher content than leaves [30]. Vasconcellos et al. [27] reported that nitrate content varied significantly among beetroot products, with the juice (12,252.90 mg/kg) showing a significantly higher concentration than chips (2031.2 mg/kg), powder (1683.50 ± 264.48 mg/kg), and cooked beetroot (1649.66 mg/kg). Verma et al. [21] showed that nitrate content might be influenced by the cultivation system, reporting that the total nitrate content was highest in the organic system (22.20 mg/100 g), while being lowest in conventional samples (15.38 mg/100 g). Moreover, other minor compounds with health benefits have been reported in beetroot. For instance, Wruss et al. [25] revealed that the oxalic acid content in beetroot is variety-dependent, with an average oxalic acid content of 412 mg/L determined for beetroot varieties. In beetroot peel extracts, 5,5,6,6-tetrahydroxy-3,3-biindolylindane has been reported, whereas the aerial parts of *B. vulgaris* cicla contain the norisoprenoid compounds (+)-dehydrovomifoliol and 3-hydroxy-5α,6α-epoxy-β-ionone [31]. A poly (3-hydroxyalkanoate) fraction has also been isolated from beetroot and was mainly composed of 3-hydroxybutyrate, with a number-average molecular weight (Mn) of 9124 Da and a relatively narrow polydispersity index (PDI) of 1.01 [31]. In addition, beetroot leaves contain beetins, which are virus-inducible type 1 ribosome-inactivating proteins. These proteins consist of a single polypeptide chain and exhibit varying degrees of glycosylation [32].

**Table 1:** Major bioactive compounds in beetroot and their functional properties.

Compound Class (Key Examples)	Main Sources/Location in the Plant	Typical Content Range/Notes	Principal Functional Properties	Technological/Food Uses	References
Nitrate (NO_3_^−^)	Root flesh and stems	Roots often 4–20 g/kg DW; stems 359 mg/kg FW	Vasodilation, blood pressure lowering, improved endothelial function, and possible ergogenic effects	Basis of beetroot juice supplements, “smart” NO-delivery formulations	[9,10,11,19,24,30]
Betalains (betacyanins: betanin, isobetanin; betaxanthins: vulgaxanthin I/II)	Especially peel and outer tissues; stems rich; also leaves	Total betalains 0.8–1.3 g/L juice; 120 mg/100 g FW in root; stems 0.58 mg/g DW	Potent antioxidant, anti-inflammatory, anti-lipidemic, anticancer, hepatoprotective, antimicrobial; contributes to gut and GI health	Natural colorant (E162) in dairy, meat, confectionery; functional ingredients, nanoformulations, peel/stem flours	[7,8,9,10,13,19,20,24,30,33,34,35]
Phenolic acids and flavonoids (gallic, protocatechuic acids; kaempferol derivatives)	Roots, leaves, and stems; high in fermented juices and by-products	Total phenolics 200–760 mg GAE/100 g (juices); biscuits 18–32 mg/100 g gallic acid, 17–25 mg/100 g protocatechuic acid	Antioxidant, anti-inflammatory, anti-carcinogenic; supports cardiometabolic and gastrointestinal health	Stability in many processing formats (juice, gels, baked goods); contributes to color and flavor	[10,13,19,20,24,33,36]
Carotenoids	Roots and leaves, enriched in juices and fermented products	Moderate; part of the total pigment pool	Antioxidant, potential anti-carcinogenic and hepatoprotective effects	Support color hue and oxidative stability in juices and snacks	[7,8,13,20]
Dietary fiber and pectic-oligosaccharides	Root (cell wall), leaves, and stems; pectin and derived oligosaccharides	Leaves and stems each 30–32 g/100 g fiber DW 9; pectic-oligosaccharides variable	Prebiotic, bifidogenic effects; increase SCFA production; support gut barrier and GI health	Flours from leaves/stems, peel powders; texture and water-binding in bakery/snacks	[20,30,33,34,36,37]
Vitamins (C, B-group, folate) and minerals (K, Mg, Fe, etc.)	Roots, juices, fermented juices; also leaves/stems	Juice: vitamin C 4–20 mg/100 mL; folate 80–109 μg/100 mL; high K, Fe, and Mg	Antioxidant defense, homocysteine metabolism, cardiometabolic support	Nutrient enrichment of beverages, snacks, probiotic/fermented products	[11,13,19,20,38,39]

### 2.4. Bioactivities

Beetroot ranks among the top vegetables for antioxidant activity, largely due to betalains plus phenolics and vitamin C [19,20,23,24]. Fermented juices, peel flours, and by-product extracts retain high antioxidant and radical-scavenging capacity [20,34,36,40]. Beetroot showed anti-cardiovascular effects through nitrate, as the nitrate-derived NO improves endothelial function, lowers blood pressure, and displays hypotensive effects that are stronger in hypertensive or overweight adults [9,10,19]. Moreover, it exhibited metabolic effects, as it showed evidence for improved glycemic responses, lipid-lowering, anti-obesity, and antidiabetic actions [10,19,35]. Betalains showed anti-inflammatory, chemopreventive, hepatoprotective, and potential antitumor activities in vitro and in vivo. Beetroot peel flour pigments showed chemoprotective and anti-clonogenic effects on cancer cells [8,12,24,34]. Renal and vascular protection: Beetroot-based interventions demonstrate vasodilatory and possible renoprotective effects in clinical contexts, including kidney disease [10,19,38]. Beetroot is exceptionally rich in betalain pigments, nitrate, phenolics, vitamins, minerals, and fiber, with important contributions also from leaves, stems, and peels. These compounds underpin strong antioxidant, anti-inflammatory, and vasodilatory effects, with promising evidence for benefits in blood pressure, cardiometabolic health, and cancer chemoprevention, though some human data remain inconsistent and mechanism-specific. As a stable, safe natural colorant and functional ingredient, beetroot and its by-products are increasingly exploited in juices, fermented beverages, baked goods, snacks, and encapsulated extracts.

Beetroot bioactives exhibit markedly different degradation patterns depending on their chemical structure and environmental sensitivity. Betalains, particularly betacyanins such as betanin, are among the most labile constituents and undergo hydrolysis, isomerization, dehydrogenation, decarboxylation and oxidative reactions under thermal, photochemical and unfavorable pH conditions. Betanin hydrolysis can generate betalamic acid and cyclo-DOPA-5-O-glucoside, whereas thermal and oxidative reactions may produce neobetanin and other dehydrogenated or decarboxylated derivatives, ultimately resulting in chromatic deterioration [41,42]. Phenolic compounds display more heterogeneous behavior, involving oxidation, hydrolysis, polymerization and transformation between bound, glycosylated, and aglycone forms; consequently, changes in total phenolic content do not necessarily represent direct degradation of all individual phenolics. Indeed, beetroot juice stored at ambient temperature exhibited reductions in phenolic content and antioxidant activity, whereas these parameters were comparatively preserved under refrigerated and frozen conditions [43]. Dietary nitrate follows a distinct stability pathway, with substantial losses occurring during prolonged storage at ambient temperature, while refrigeration and freezing markedly improve retention [43,44]. Other components, including ascorbic acid and carotenoids, are primarily susceptible to oxidative and thermally induced transformations. Overall, the degradation of beetroot bioactives should therefore be considered as a dynamic network of hydrolytic, oxidative, enzymatic and thermally induced reactions rather than simple disappearance of the parent compounds. These mechanisms provide a strong scientific basis for the development of encapsulation and nanocarrier systems capable of protecting beetroot bioactives from oxygen, light, temperature and unfavorable gastrointestinal conditions.

## 3. Limitations in Conventional Delivery Systems of Beetroot Bioactives

Conventional delivery systems for beetroot bioactive compounds are associated with several limitations that hinder their effective utilization. These include low solubility and bioavailability, rapid degradation under gastrointestinal conditions, and unfavorable interactions with food matrices that reduce their functional performance. Additionally, the poor stability of these compounds during storage leads to significant losses in color, antioxidant capacity, and overall quality. Collectively, these challenges limit the industrial applicability of beetroot-derived bioactives and highlight the need for advanced delivery systems such as nanoencapsulation to enhance their stability, bioavailability, and functional efficiency.

### 3.1. Chemical Instability During Processing and Storage

Betalains are heat-, pH-, oxygen-, and light-sensitive, leading to rapid degradation in juices, jams, and dried products [8,45,46]. Beetroot betalains are particularly vulnerable to thermal processing, with degradation involving reactions such as hydrolysis of the aldimine linkage, decarboxylation, dehydrogenation, isomerization, and formation of secondary products such as betalamic acid and related derivatives [47,48]. The conventional drying (simple convection) markedly decreases betalains and polyphenols versus optimized or hybrid drying, while thermal shock + microwave preserved pigments better than single-step hot air [49,50]. The traditional home processing (boiling, pickling, simple juicing) causes large losses in total phenolics and antioxidant capacity; only a small fraction remains bioaccessible after digestion [45]. In vitro digestion showed that only a fraction of flavonoids and betalains became dialyzable and potentially absorbable, while much remains bound to the matrix or degraded in gastric/intestinal conditions [39,45]. Glycosylated betalains can be hydrolyzed by digestive enzymes and microbiota, further reducing intact compound availability and making pharmacokinetics unpredictable [39]. In addition, many phytochemicals suffer from chemical instability, and low membrane permeability, leading to low systemic exposure when delivered in conventional formulations [51,52]. Conventional beetroot delivery systems (boiled, dried, juices, simple supplements) are limited by instability, low bioaccessibility, and sensory and shelf-life constraints, meaning that only a modest portion of beetroot bioactives reaches systemic circulation.

### 3.2. Limited Protection During Gastrointestinal Digestion

Conventional delivery systems provide little or no physical protection against the highly dynamic gastrointestinal environment. Following ingestion, beetroot bioactives encounter salivary enzymes, gastric acid, digestive enzymes, bile salts, changing ionic strength, and intestinal pH [53,54]. Consequently, a compound that remains stable during storage may undergo substantial degradation after ingestion. This is particularly relevant for betanin because its hydrophilic nature does not provide the type of physicochemical protection associated with lipid-soluble compounds incorporated into suitable delivery matrices. Experimental digestion studies support this limitation. In a simulated gastrointestinal digestion study, purified betanin showed only a relatively small loss during the oral phase but declined to approximately 65% of its initial amount after simulated gastric digestion and approximately 46% after the small-intestinal phase; no intact betanin was detected after subsequent ex vivo colonic fermentation [55]. Similarly, a study investigating the gastrointestinal fate of *B. vulgaris* constituents reported that betanin was highly unstable during digestion and that this instability was accompanied by a reduction in antioxidant and antihemolytic activities [56]. These observations indicate that the chemical stability of the ingested compound and its bioaccessibility are not equivalent parameters. Processing may disrupt plant tissues and improve release, but excessive thermal or oxidative treatment may simultaneously destroy susceptible compounds. For example, freezing of beetroot altered matrix structure and increased the release of total phenolics and antioxidant activity during simulated gastric digestion compared with fresh beetroot, illustrating that processing can either enhance or restrict bioaccessibility depending on the nature of the treatment and matrix [57,58].

### 3.3. Low and Variable Bioaccessibility

Another critical limitation is the relatively low and variable fraction of beetroot bioactives that becomes available for intestinal absorption. Bioaccessibility refers to the fraction released from the food matrix and remaining available for absorption in the gastrointestinal tract, whereas bioavailability additionally requires absorption and subsequent appearance in the systemic circulation in an available form [59]. Clifford et al. [60] administered either beetroot juice or whole beetroot to healthy volunteers and found that, despite relatively high betanin intake, intact betanin was not detectable in plasma during the 8-h sampling period. In contrast, nitrate-derived NOx increased following consumption. This finding is important because it demonstrates that a food can produce measurable physiological effects without necessarily generating substantial circulating concentrations of the originally ingested betalain. The bioaccessibility of betalains in beetroot juice obtained under high-pressure techniques was investigated [54]. A decrease in betacyanins of 22% was observed during simulated oral digestion, and the content of these compounds remained at a similar level in the gastric digestion stage, whereas a significant decrease was noted after the intestinal digestion step (the smallest decreases were 44% and 74%, compared to the gastric digestion stage) [54]. Regarding betaxanthins, the conditions prevailing at the stage of simulated mouth digestion did not adversely influence the stability of these compounds. However, at the gastric digestion step, a significant decrease was observed (av. 70%) compared to the oral digestion stage. As a result of simulated intestinal digestion, betaxanthins were further degraded by 17% [54].

## 4. Nanocarriers of Beetroot Bioactives and Applications

**Figure 2:**
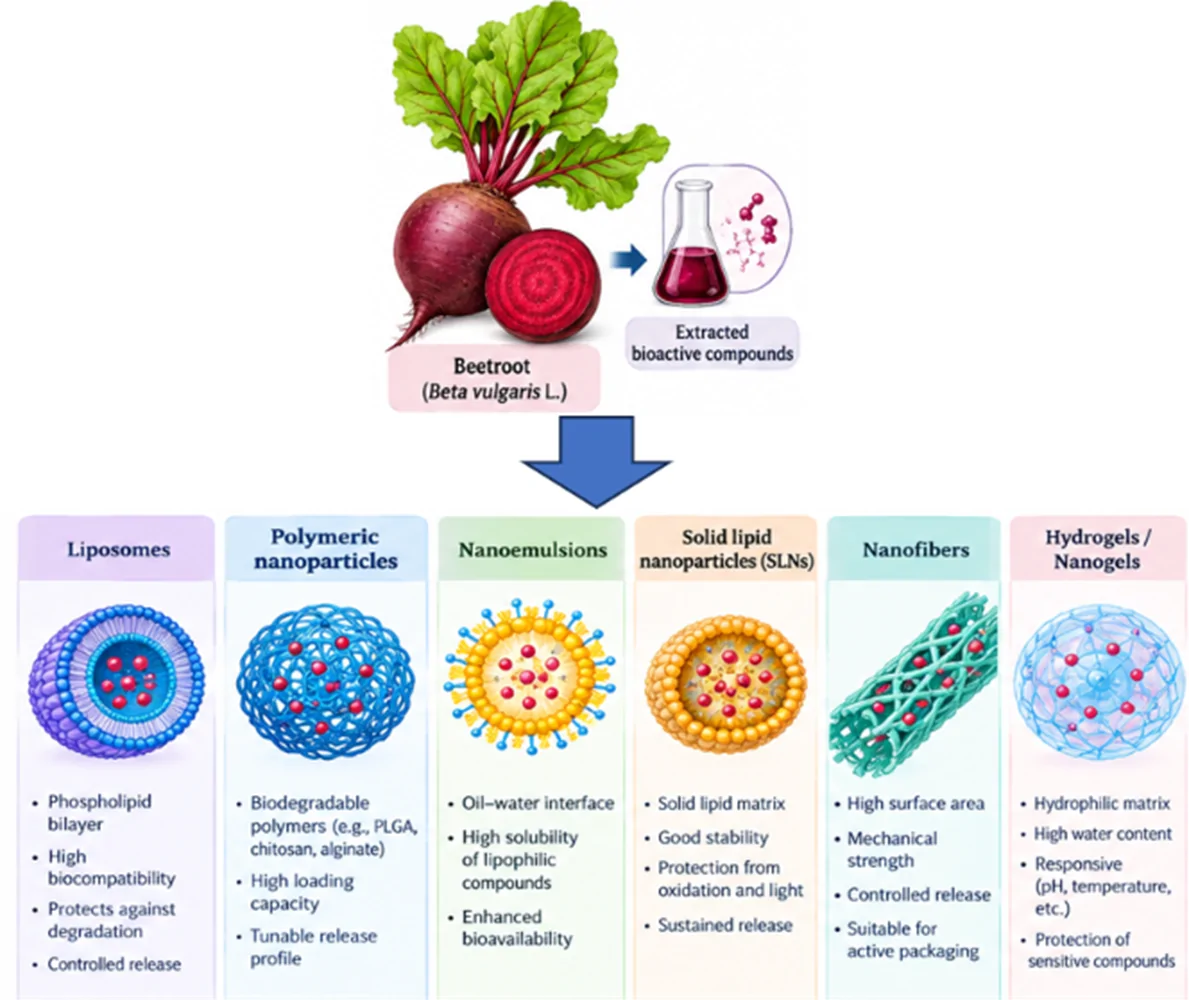
Different nanocarriers for the delivery of beetroot bioactive compounds.

Nanocarriers such as liposomes, nanoemulsions, and polymeric nanoparticles are essential for unlocking the full potential of beetroot bioactives by enhancing stability, bioavailability, and functional performance, enabling their use across food, pharmaceutical, and biomedical sectors (Table 2 and Figure 2).

**Table 2:** Nanocarriers for Encapsulation of Beetroot Bioactives.

Nanocarrier Type	Composition/Material	Encapsulated Beetroot Bioactives	Key Findings	Key Advantage	Limitations	Typical Applications	References
Liposomes	Lipids of L-α-phosphatidylcholine from soybean and cholesterol	Beetroot juice	Anticancer activity and liposomal beetroot pigments could serve as green photosensitizers	Dual encapsulation capability	Cost and stability issues	Pharmaceutical and food systems	[61]
Liposomes	Soylecithin, vit. C, distilled water, and star anise oil.	Iron, betalain, and other bioactive compounds	Enhanced the bioavailability of iron and betalain and demonstrated a protective effect on other bioactive compounds	Dual encapsulation capability	Cost and stability issues	Pharmaceutical and food systems	[62]
Solid lipid nanoparticles	Glyceryl monostearate, soya lecithin, and Poloxamer 407	Betacyanin extract	Showed a sustained release of 91.73% over 8 h and demonstrated moderate antidiabetic activity	Oxidative protection, stability	Low loading capacity	Functional foods	[63]
Self-nanoemulsifying drug delivery systems	Olive, linseed, castor, and sesame oils (oil phase), Tween^®^ 20 (Tw20), Tween^®^ 80 (Tw80) (surfactants (SAs)), dimethyl sulfoxide (DMSO), and propylene glycol (PG) (co-surfactants (Co-SAs))	Beet leaf extract	Showed hepatoprotective activity	Enhanced Solubility and Bioavailability	Limited drug loading capacity and stability issues	Pharmaceutical, cosmeceutical, and food Applications	[64]
Nanoemulsions	Water-in-oil (W/O) and polyglycerol polyricinoleate	Betalain extract	Color and particle size stability, as well as high antioxidant activity	Improved solubility, bioavailability	Physical instability	Pharmaceutical, cosmeceutical, and food Applications	[65]
Nanogel	Chitosan and tripolyphosphate	Betanin	Showed mucoadhesive photoprotective, pH stability, and antioxidant effects, as well as absence of cytotoxicity, and protective effect against injury agents	High loading capacity, excellent biocompatibility, and biodegradability	Limited mechanical strength, premature release, or leakage	Pharmaceutical, cosmeceutical, and food Applications	[66]
Nanocomposite	Chitosan, pentasodium tripolyphosphate, and ethanol	Beetroot extract	Antioxidant, anticancer, and anti-inflammatory properties	Biodegradability and biocompatibility	Poor water resistance	Pharmaceutical, cosmeceutical, environmental, and food Applications	[67]
Nanoemulsions	Gelatin, cress seed gum, polyglycerol polyricinoleate 4150, and sunflower oil	Red beetroot extract	Showed positive effects on the physicochemical and rheological properties	Improved solubility, bioavailability	Physical instability	Pharmaceutical, cosmeceutical, and food Applications	[68]
Nanofiber	Kafirin and polyethylene oxide	Red beetroot extract	Color stability	High surface area, sustained release	Moisture sensitivity	Active packaging	[69]
Nanofiber	Zein	Red beetroot extract	Color change indication through pH change	High surface area, sustained release	Moisture sensitivity	Active packaging	[70]
Nanofiber	Vapochromic polylactic acid	Betalains	Green and sustainable sensor for toxic ammonia	High surface area, sustained release	Moisture sensitivity	Active packaging	[71]

### 4.1. Lipid-Based Nanocarriers

Solid lipid nanoparticles (SLN) and nanostructured lipid carriers (NLC) are widely used to protect lipophilic bioactives, improve solubility, permeability and bioavailability, and enable controlled release [8,72]. Liposomes, nanoemulsions and self-emulsifying systems (SEDDS) are established for vitamins and carotenoids and are conceptually applicable to beetroot lipophilic constituents co-delivered with oils [72,73,74]. Phytosomes (phytochemical-phospholipid complexes) enhance the penetration and bioavailability of plant polyphenols, especially for topical or barrier-crossing delivery [75,76]. Fadeel et al. (2024) prepared beetroot juice, betanin, liposomal-loaded beetroot juice, and liposomal-loaded betanin and tested them as photosensitizers on normal (HEK 293) and human lung cancer (A549) cell lines. Liposomes loaded with beetroot juice and with betanin were prepared by the reverse-phase evaporation technique by mixing lipids of L-α-phosphatidylcholine from soybean and cholesterol at a 10:1 *w*/*w* ratio. The irradiation significantly enhanced the cytotoxicity of liposomes loaded with beetroot juice and with betanin on HEK 293 cells and A549 cells [61]. These results proved the positive effect of light and liposomal encapsulation on the anticancer activity of betanin and beetroot juice, suggesting the efficiency of liposomal beetroot pigments as green photosensitizers [61]. Kaushik et al. [63] developed SLNs containing betacyanin extracted from red beetroot using heat homogenization and ultrasonication, with glyceryl monostearate, soya lecithin, and Poloxamer 407 as core ingredients. The SLNs showed a sustained release of 91.73% over 8 h and demonstrated moderate antidiabetic activity with an IC_50_ value of 23.25 μg/mL. These findings suggest that betacyanin-loaded SLNs hold significant promise as a stable and effective alternative for controlled drug delivery in diabetes management [63]. Sheir et al. [62] investigated the efficacy of vitamin C encapsulation in liposomes for improving iron bioavailability and preserving bioactive compounds in sweet-sour beetroot sauce. The liposome was formed by combining soy lecithin and vit. C at a 1:1 ratio, distilled water, and star anise oil. The results demonstrated that encapsulation significantly enhanced the bioavailability of iron and betalain and demonstrated a protective effect on other bioactive compounds [62]. Kassem et al. [64] studied the development and optimization of self-nanoemulsifying drug delivery systems (SNEDDSs) to enhance the hepatoprotective activity of beet leaf extract. SNEDDSs were constituted of olive, linseed, castor, and sesame oils (oil phase), Tween^®^ 20 (Tw20) and Tween^®^ 80 (Tw80) (surfactants (SAs)) as well as dimethyl sulfoxide (DMSO) and propylene glycol (PG) (co-surfactants (Co-SAs)). The beet leaf extract-SNEDDSs showed hepatoprotective activity in a thioacetamide-induced hepatotoxicity rat model, which promoted liver functions, inflammatory markers, and histopathological findings [64]. The results indicated that SNEDDS is a potential nanocarrier system that improves the hepatoprotective activity of the beet leaf extract [64]. Different nanocarriers offer unique advantages depending on the targeted application (food, pharmaceutical, or packaging). Lipid-based and biopolymer-based nanocarriers are particularly promising for beetroot bioactives due to their safety and compatibility with food systems. Another nanoemulsion was prepared by loading the betalain extract of beetroot in water-in-oil (W/O) and polyglycerol polyricinoleate (PGPR) at a concentration of 4.0% as a hydrophobic surfactant for the Sacha Inchi oil [65]. The resulting bioactive nanoemulsion showed color and particle size stability within a pH range of 3.0 to 9.0 at high temperatures up to 90 °C, and exhibited high antioxidant activity [65].

Nanoliposomes are particularly appropriate for betalains because their aqueous core can accommodate highly water-soluble pigments while the phospholipid bilayer provides protection against oxygen, light, and thermal stress. This advantage is supported by a study demonstrating improved stability and biological performance of betalain-loaded liposomes; notably, betalain liposomes enhanced antioxidant-related effects following ingestion [77]. However, phospholipid oxidation, relatively high production cost, and limited physical stability remain important limitations. Nanoemulsions, particularly oil-in-water systems, are more appropriate for relatively lipophilic beetroot constituents and moderately hydrophobic phenolics, because the oil phase provides a solubilizing reservoir and the nanoscale droplets facilitate digestion and micellar incorporation [78]. For betalains, conventional O/W nanoemulsions are less intrinsically suitable because betalains preferentially partition into the aqueous phase. Nevertheless, double nanoemulsions can overcome this limitation by providing an internal aqueous compartment. A recent beetroot study produced betalain-loaded double nanoemulsions of approximately 94 nm, with good stability over pH 3–9 and enhanced antioxidant activity [65]. SLNs provide a crystalline lipid matrix capable of protecting oxidation-sensitive, relatively lipophilic phenolics and enabling sustained release [79]. Their major drawback is potential expulsion of bioactives during lipid crystallization. NLCs partially overcome this problem through incorporation of liquid lipids, producing a less ordered matrix with greater loading capacity and improved retention [80]. Thus, NLCs may be particularly advantageous for beetroot phenolics with limited aqueous solubility. Overall, liposomes and double nanoemulsions are more rational for betalains, whereas NLCs/SLNs are better suited to hydrophobic or poorly water-soluble phenolics.

### 4.2. Polymer-Based Nanoencapsulation

Nanoencapsulation with food-grade polymers and biopolymers is emerging to stabilize these bioactives and enable controlled delivery. Silva Nieto et al. [66] encapsulated beetroot extract within a chitosan-based nanogel designed via ionic crosslinking with tripolyphosphate (TPP) for betanin delivery, mainly in the ophthalmic environment. The results revealed that the beetroot extract-chitosan-based nanogel showed mucoadhesive photoprotective, pH-stability, and antioxidant effects [66]. Moreover, in vitro assays on beetroot extract and chitosan-based nanogel using human retinal cells showed the absence of cytotoxicity and demonstrated a protective effect against injury agents (LPS and H_2_O_2_), making beetroot extract and chitosan-based nanogel a promising platform for ophthalmological use [66]. El-ghannam et al. [67] designed a beetroot extract and chitosan-based nanocomposite and investigated its effect on human epithelial colorectal adenocarcinoma (Caco-2), human epithelial ductal breast carcinoma (T-47D), and human epithelial lung carcinoma (A549) cells. The beetroot extract and chitosan-based nanocomposite were prepared by mixing the acidic chitosan solution with the beetroot extract under magnetic stirring for 1 h at room temperature, and then centrifuging, washing, and drying. The application of beetroot extract and chitosan-based nanocomposite successfully eradicated 89% of A549, 88% of T-47D, and 83% of Caco-2 cell lines at a dose of 250 μg/mL [67]. These findings revealed that beetroot extract displayed antioxidant and anti-inflammatory properties with the antiangiogenic mechanism and inhibition of tumors by chitosan nanoparticles, resulting in a unique and innovative strategy for cancer treatment [67]. In a study, beet leaves, a rich source of phenolics and natural pigments, were valorized into a nanocarrier system to enhance antioxidant protection and controlled release [81]. The nanocarrier was formed with chitosan, cellulose nanocrystals, and beet leaf extract encapsulated in mesoporous silica nanoparticles. The film formed from the nanocarrier effectively limited oxidative and microbial spoilage, maintaining meat quality by moderating pH, reducing oxidation, and suppressing microbial growth below spoilage thresholds during 8-day refrigerated storage, suggesting a multifunctional packaging system that offers a scalable, eco-friendly solution for meat protection through synergistic integration of natural bioactives and nanotechnology [81]. In another study, beetroot juice was encapsulated using nanopowder of tragacanth gum hydrogel, followed by freeze-drying [82]. The resulting nano-nutraceuticals naturally contain essential nutrients such as iron, folic acid, ascorbic acid, chlorophyll, niacin, and sugars, without the need for chemical processing or preservatives [82]. Ghoorchi et al. [68] nanoencapsulated the red beetroot extract based on betanin with gelatin and cress seed gum. To prepare the nanocarrier, a W/O emulsion was formed using a 30% (*w*/*v*) red beetroot extract solution and sunflower oil, along with 3% of the lipophilic emulsifier polyglycerol polyricinoleate 4150, which was transformed into a W/O/W emulsion by adding gelatin and cress seed gum solutions as a hydrophilic emulsifier. The results showed that the nanoencapsulation had positive effects on the physicochemical and rheological properties of the nanocapsules [68].

Chitosan nanogels/nanoparticles are particularly appropriate for betanin-rich beetroot extracts because betanin and other betalains are highly hydrophilic and readily interact with protonated amino groups of chitosan. Ionic crosslinking with tripolyphosphate can generate nanoscale, mucoadhesive carriers and provide protection against light and pH-induced degradation. Indeed, chitosan nanogels containing beetroot extract exhibited a mean size of 166 nm, 45% encapsulation efficiency, and biphasic, sustained release, demonstrating their potential for betanin delivery [66,83]. However, chitosan systems can exhibit relatively low loading efficiencies and aggregation at unfavorable pH conditions, requiring careful control of molecular weight, degree of deacetylation, and crosslinking density. Alginate-based nanoparticles or ionically gelled carriers are likewise attractive for betalains because their hydrophilic polysaccharide network can entrap water-soluble pigments under mild, food-compatible conditions. Beetroot extract encapsulated in alginate showed markedly different efficiencies depending on gelation strategy, illustrating that the processing route is as important as polymer identity [84]. Nevertheless, alginate matrices can be highly water-sensitive and may exhibit relatively rapid swelling or release under gastrointestinal conditions. Furthermore, pectin can provide an acidic, anionic environment and gastrointestinal-responsive release, while combination with proteins or chitosan can produce denser networks. Beetroot betalain-loaded albumin–pectin cryogels achieved 88–97% encapsulation efficiency and retained substantial betalain and phenolic fractions during storage, supporting the value of polysaccharide–protein/polymer hybrid systems [85,86].

### 4.3. Nanofibers as Carriers of Beetroot Bioactives

Nanofibers have emerged as an effective delivery system for stabilizing and enhancing the functionality of beetroot bioactive compounds, particularly betalains, and polyphenols. These compounds are highly sensitive to environmental factors such as light, oxygen, and temperature, leading to rapid degradation during processing and storage. Electrospinning is the most commonly used technique to fabricate nanofibers, enabling the encapsulation of beetroot extracts within biopolymeric matrices such as chitosan, gelatin, or polyvinyl alcohol. This approach improves the stability, solubility, and controlled release of bioactives. Additionally, nanofibers offer a high surface-area-to-volume ratio, enhancing bioavailability and functional performance. In food and packaging applications, beetroot-loaded nanofibers can serve as antioxidant-rich coatings or active packaging materials, extending shelf life and improving product quality. Intelligent electrospun pH indicators were produced from bio-nanocomposite kafirin-polyethylene oxide (PEO) containing red beetroot extract [69]. The bio-nanocomposite was obtained by combining kafirin and PEO at a ratio of 25% and 2%, respectively. The results showed that the color of the indicators was significantly reversible after pH changes, with highly desirable reversibility observed at pH values of 1, 3, 4, 5, 7, 9, and 10, proving that the red beetroot extract-loaded bio-nanocomposite pH indicator accompanied by evaluation of color characteristics through image processing technique, can serve as a time-efficient, accurate tool for detecting and tracking pH changes caused by food spoilage [69]. In a study, smart nanofibers were produced by blending zein with betalains extracted from beetroot and applied for food preservation [70]. The spoilage monitoring results on black grapes with zein and beetroot extract-based nanofibers showed a significant color change indication through pH change, making them new pH-sensitive materials that can be used as halochromic indicators for monitoring black grape spoilage [70]. In another study, vapochromic polylactic acid was combined with betalains extracted from beetroot to produce nanofibers usable as a green and sustainable sensor for toxic ammonia [71]. Overall, nanofiber-based systems represent a promising strategy for preserving and delivering beetroot bioactives in functional foods and nutraceutical applications.

## 5. Encapsulation Efficiency, Stability, and Release Behavior

Encapsulation and nanoencapsulation have emerged as important strategies for improving the physicochemical stability, gastrointestinal protection, and potential bioavailability of beetroot-derived betalains. Encapsulation efficiency (EE) is a critical parameter in delivering beetroot bioactives, particularly betalains and phenolics, as it determines the proportion successfully entrapped within nanocarriers such as liposomes, nanoemulsions, or polymeric nanoparticles [87,88]. High EE enhances protection against environmental stressors like light, oxygen, and pH fluctuations, thereby improving stability during processing and storage [89]. Stability studies often show that encapsulation significantly reduces degradation and color loss [90]. Release behavior is influenced by carrier composition and environmental conditions, enabling controlled or targeted delivery in gastrointestinal systems [66,91]. The spray-drying of beet by-product phenolics with polyvinylpyrrolidone yields 60–89% process yield and phenolic load of 136 and 1026 mg gallic acid equivalents/g [40]. The ready-to-eat beetroot soup microencapsulated by lyophilization with starch or maltodextrin showed good yields (75–89%) and traps >50% of betalains in microcapsules [92]. Cookies fortified with pumpkin-protein-encapsulated beetroot juice polyphenols minimized the degradation of the bioactive compounds, as the phenolic content in cookies was almost the same without significant degradation throughout the whole storage period, while the betalain content was slightly decreased by 20%. [93]. Encapsulation shields beet phenolics and betalains from light, oxygen, and water activity, extending shelf life and preserving antioxidant properties [40,92,94]. For analogous plant bioactives, nano/microcarriers (starch nanoparticles, alginate beads, Pickering emulsions, zein- or polysaccharide-based particles) commonly showed slow release in gastric conditions and higher release/bioaccessibility in intestinal media, preserving antioxidant activity [95,96,97]. Encapsulating beetroot bioactives using polysaccharides (PVP, starch, maltodextrin) or protein–polysaccharide systems can achieve high encapsulation efficiency, markedly improve light/thermal/storage stability, and enable controlled gastrointestinal release. Patterns from beetroot and analogous plant bioactives suggest targeting minimal release in the stomach and enhanced intestinal release, while integrating encapsulates into stable food matrices (soups, baked goods) to preserve betalains, and phenolics.

Amjadi et al. [98] reported an EE of 80.35 ± 1% for betanin-loaded nanoliposomes, demonstrating the high affinity of the liposomal aqueous compartment for betanin. A subsequent study by the same research group reported betanin-loaded nanoliposomes with a particle size of approximately 36–40 nm, a ζ-potential of approximately −19 mV, and a loading capacity of approximately 26%. Liposomal nanoencapsulation resulted in a slower release profile under simulated gastrointestinal conditions and improved betanin stability and antioxidant activity during digestion [98]. Likewise, it was reported that betanin-loaded nanoliposomes had a particle size of approximately 36–40 nm, a ζ-potential of approximately −19 mV, and a loading capacity of approximately 26% [99]. Nanoliposomal encapsulation resulted in a slower release profile under simulated gastrointestinal conditions and improved betanin stability and antioxidant activity during digestion [99]. A further development is the incorporation of polymer coatings onto liposomes. Chitosan-coated nanoliposomes increased in size from approximately 156.4 nm for uncoated vesicles to 217.2 nm at 1% chitosan, while the uncoated system exhibited PDI 0.27, ζ-potential −19.5 mV, EE 42.7%, and LC 2.6% [100]. Chitosan coating reduced the release of betanin in aqueous medium from 29.0% to 15.8%, indicating that the polymer layer acts as an additional diffusion barrier. The coated liposomes remained relatively stable under simulated oral and gastric conditions and underwent substantial structural changes in simulated intestinal fluid, presumably owing to bile salts and digestive enzymes [100]. Thus, chitosan coating sacrifices some of the size and loading advantages of uncoated nanoliposomes but provides improved control over premature release. An even more sophisticated multilayer system consisting of chitosan- and chondroitin sulfate-coated liposomes exhibited a progressive increase in particle size from 223.57 to 522.33 nm, PDI from 0.22 to 0.35, and ζ-potential from −27.73 to +16.70 mV as polymer layers were introduced [91]. Importantly, the multilayer system exhibited slower betanin release and greater stability during simulated digestion than uncoated or single-layer systems [91]. These findings indicate that multilayer nanoliposomes may be particularly advantageous where prolonged gastrointestinal protection is desired.

A beetroot-extract-loaded chitosan–tripolyphosphate nanogel exhibited a particle size of 166 ± 6 nm, PDI of 0.30 ± 0.03, ζ-potential of +28 ± 1 mV, and EE of 45 ± 3% [66]. The positive surface charge is particularly relevant because it promotes electrostatic interaction with negatively charged mucin, potentially increasing residence time at biological interfaces. Release was biphasic, consisting of an initial burst followed by a slower sustained phase over approximately 12 h. The system also provided photoprotection and pH stabilization of the beetroot extract [66]. Compared with nanoliposomes, therefore, chitosan nanogels generally provide moderate EE but stronger mucoadhesive characteristics and potentially more sustained release. Nevertheless, the beetroot nanogel study was primarily designed for ophthalmic delivery and did not provide a complete standardized oral gastrointestinal bioaccessibility assessment. Consequently, claims regarding improved oral bioavailability should be distinguished from demonstrated physicochemical stability and controlled release. Nanoemulsions are attractive, particularly for the incorporation of beetroot bioactives into beverages and other aqueous food matrices. Tran et al. [65] developed a double nanoemulsion containing beetroot betalain extract, obtaining a mean particle size of 93.8 nm by dynamic light scattering and 82.3 nm by transmission electron microscopy. The formulation retained relatively stable particle size and color over pH 3–9 and temperatures up to 90 °C and remained stable for approximately 24 days at room temperature and >30 days under refrigerated conditions [65]. The principal advantage of this system is therefore its small particle size combined with relatively good physicochemical stability. However, the study did not provide the same depth of gastrointestinal release and bioaccessibility information available for the betanin nanoliposome studies. This highlights an important methodological gap: a small hydrodynamic diameter should not automatically be interpreted as evidence of enhanced intestinal absorption or bioaccessibility. Overall, nanoliposomes, polymer-coated liposomes, chitosan nanogels and nanoemulsions represent promising platforms for improving the stability and gastrointestinal performance of beetroot betalains. Nanoliposomes can achieve very small particle sizes and relatively high loading capacities, whereas polymer-coated systems provide additional control over premature gastrointestinal release. Chitosan nanogels offer mucoadhesive and sustained-release characteristics, while double nanoemulsions provide excellent nanoscale dispersion and physicochemical stability. Nevertheless, direct comparisons remain difficult because studies differ substantially in the betalain source, carrier composition, encapsulation procedure, storage conditions, and gastrointestinal digestion model. Moreover, high encapsulation efficiency does not necessarily correspond to high bioaccessibility. Future investigations should therefore adopt standardized simulated gastrointestinal protocols and quantify individual betalains, particularly betanin and isobetanin, in the soluble and micellar intestinal fractions. Integrating particle-size evolution, encapsulation efficiency, loading capacity, storage retention, gastrointestinal release, and intestinal bioaccessibility would provide a more meaningful assessment of the nutritional performance of beetroot nanocarriers.

## 6. Current Challenges and Future Perspectives in Nanoencapsulation of Beetroot Bioactive Compounds

Nanoencapsulation has emerged as a promising strategy to enhance the stability, bioavailability, and controlled delivery of beetroot-derived bioactive compounds, particularly betalains, and phenolics. Despite significant advances, several scientific, technological, and regulatory challenges remain, limiting the translation of laboratory findings into industrial and commercial applications. Addressing these challenges is essential to fully exploit the functional and therapeutic potential of beetroot bioactives.

### 6.1. Intrinsic Instability of Beetroot Bioactive Compounds

One of the primary challenges in nanoencapsulation of beetroot bioactives lies in the inherent instability of these compounds. Betalains, the main pigments in beetroot, are highly sensitive to environmental factors such as pH, temperature, light, and oxygen [88]. Their degradation during processing, storage, and digestion significantly reduces their antioxidant capacity and functional efficacy. Although nanoencapsulation aims to protect these compounds, achieving complete stabilization remains difficult. Even encapsulated systems may undergo degradation due to imperfect barrier properties of the carrier materials or exposure to harsh processing conditions. For instance, studies have shown that encapsulation may still result in reduced antioxidant activity compared to native extracts due to structural alterations during formulation [65]. Therefore, there is a need for advanced nanocarriers with superior protective capabilities, particularly those resistant to thermal processing and oxidative environments. Hybrid systems combining biopolymers, lipids, and antioxidants could be explored to enhance stability.

### 6.2. Low Encapsulation Efficiency and Loading Capacity

Achieving high encapsulation efficiency and loading capacity is a persistent challenge. The physicochemical properties of beetroot compounds, especially their hydrophilic nature (e.g., betalains), complicate their incorporation into many nanocarriers designed for lipophilic compounds. Encapsulation efficiency depends on multiple factors such as carrier material, preparation method, and process parameters. Variations in particle size, polydispersity, and surface charge can lead to inconsistent loading and release profiles [89]. Additionally, interactions between the core material (beetroot extract) and the wall matrix may lead to leakage or degradation during storage [89]. More systematic studies are required to optimize formulation parameters and understand molecular interactions between beetroot bioactives and encapsulating matrices. Computational modeling and AI-driven formulation design could accelerate this process.

### 6.3. Limited Understanding of Release Mechanisms and Bioavailability

While nanoencapsulation is designed to improve bioavailability, the actual behavior of encapsulated beetroot compounds in the gastrointestinal tract (GIT) remains poorly understood. Factors such as pH variations, digestive enzymes, bile salts, and interactions with other dietary components can significantly affect release kinetics and absorption [89]. For example, lipid-based nanocarriers may undergo structural changes in the presence of bile salts, altering the release profile of encapsulated betalains [89]. Furthermore, food matrix interactions (e.g., proteins, fibers, minerals) may hinder the release and absorption of bioactives. There is a lack of comprehensive in vivo studies and standardized in vitro digestion models specifically tailored for beetroot nanoformulations. More research is needed to correlate the physicochemical properties of nanoparticles with biological performance.

### 6.4. Challenges in Selection of Suitable Nanocarriers

Choosing appropriate nanocarriers for beetroot bioactives is complex due to the diversity of compounds with different chemical properties. Biopolymer-based systems (e.g., chitosan, alginate), lipid-based carriers (liposomes, nanoemulsions), and protein-based systems all have advantages and limitations. The compatibility between core and wall materials is critical. Poor compatibility can lead to phase separation, low stability, or premature release [89]. Additionally, some carriers may not provide sufficient protection against environmental stressors such as heat or oxidation. The development of multifunctional and stimuli-responsive nanocarriers that can adapt to different environmental conditions and target specific release sites is still in its infancy. Comparative studies evaluating different carrier systems for beetroot compounds are also limited.

### 6.5. Processing Constraints and Scalability Issues

Most nanoencapsulation techniques, such as high-pressure homogenization, ultrasonication, electrospraying, and microfluidics, are optimized at laboratory scale. However, scaling up these processes for industrial production presents significant challenges. The high-energy methods required for nanoemulsion or liposome formation increase production costs and may degrade sensitive compounds [89]. Additionally, maintaining consistency in particle size, morphology, and encapsulation efficiency during large-scale production is difficult. The integration of nanoencapsulation into existing food processing systems (e.g., pasteurization, extrusion) is another challenge, as these processes may compromise nanoparticle integrity [89]. Thus, there is a need for cost-effective, scalable, and energy-efficient encapsulation techniques. Continuous processing technologies and green manufacturing approaches should be further explored.

### 6.6. Stability During Storage and Shelf-Life Limitations

Ensuring the long-term stability of nanoencapsulated beetroot bioactives remains a major hurdle. Environmental factors such as temperature, humidity, oxygen, and light can still induce degradation over time, even in encapsulated systems [89]. Moreover, nanoparticle aggregation, coalescence, and structural changes can occur during storage, affecting functionality and release behavior. For example, encapsulated pigments may still experience color loss or reduced antioxidant activity over time. More studies are needed on the shelf-life of nanoencapsulated beetroot products under real storage conditions. The development of packaging systems that complement nanoencapsulation (e.g., oxygen-barrier materials) is also an emerging area.

### 6.7. Safety, Toxicity, and Regulatory Concerns

The safety of nanomaterials used in food applications is a critical concern. Regulatory frameworks for nanoencapsulated food ingredients are still evolving, with significant variation across countries [89]. Consumer perception is another barrier, as skepticism toward nanotechnology in food products may limit market acceptance. Comprehensive toxicological studies and standardized regulatory guidelines are urgently needed. Transparency, labeling, and consumer education will play a key role in acceptance. While nanoencapsulation offers a powerful approach to enhance the functionality of beetroot bioactive compounds, several challenges hinder its full-scale application. These include compound instability, low encapsulation efficiency, limited understanding of bioavailability, scalability issues, storage instability, safety concerns, and lack of clinical validation.

Future research on nanoencapsulation of beetroot bioactive compounds is expected to focus on the development of smart, stimuli-responsive delivery systems capable of targeted and controlled release under specific physiological conditions. The integration of artificial intelligence tools for formulation optimization represents a promising approach to accelerate the design of efficient and stable nanocarriers. Additionally, the incorporation of nanoencapsulated beetroot bioactives into edible and bioactive packaging systems offers significant potential for enhancing food quality and safety. Emerging trends in personalized nutrition may further drive the development of tailored delivery systems adapted to individual health needs. However, achieving successful industrial translation will require addressing key challenges related to scalability, cost-effectiveness, regulatory approval, and consumer acceptance.

## 7. Conclusions

Nanoencapsulation has emerged as a promising strategy to overcome the instability, low bioavailability, and sensitivity of bioactive compounds of beetroot. This review highlights that a wide range of nanocarrier systems, including lipid-based, polymeric, and nanofiber-based platforms, have demonstrated significant potential in enhancing encapsulation efficiency, improving physicochemical stability, and enabling controlled release of key compounds such as betalains, and phenolics. The use of these nanocarriers enhanced the stability and release behavior of the bioactive compounds of beetroot. Despite these advances, several challenges remain, including issues related to large-scale production, cost-effectiveness, regulatory approval, and limited in vivo validation. Addressing these barriers is essential to bridge the gap between laboratory research and industrial application. In this context, the integration of sustainable materials, green extraction technologies, and advanced design approaches such as smart delivery systems and artificial intelligence-driven formulation is expected to play a crucial role. Overall, nanoencapsulation represents a favourable approach for unlocking the full potential of beetroot bioactives in functional foods, nutraceuticals, and bioactive packaging systems. Future research should prioritize scalability, safety assessment, and real-world application to ensure successful commercialization and broader societal impact.

## Data Availability

No new data were created or analyzed in this study.

## References

[1] Câmara J.S., Albuquerque B.R., Aguiar J., Corrêa R.C.G., Gonçalves J.L., Granato D., Pereira J.A.M., Barros L., Ferreira I.C.F.R. (2020). Food Bioactive Compounds and Emerging Techniques for Their Extraction: Polyphenols as a Case Study. Foods.

[2] Kris-Etherton P.M., Hecker K.D., Bonanome A., Coval S.M., Binkoski A.E., Hilpert K.F., Griel A.E., Etherton T.D. (2002). Bioactive compounds in foods: Their role in the prevention of cardiovascular disease and cancer. Am. J. Med..

[3] Garza-Juárez G., Pérez-Carrillo A., Arredondo-Espinoza E., Islas E.U., Benítez-Chao J.F., Escamilla-García D.F., Nutraceuticals E., Elizabeth R., Cerda B., Cruz-Hernández M.A. (2023). Nutraceuticals and Their Contribution to Preventing Noncommunicable Diseases. Foods.

[4] Samtiya M., Aluko R.E., Dhewa T., Moreno-Rojas J.M. (2021). Potential Health Benefits of Plant Food-Derived Bioactive Components: An Overview. Foods.

[5] Zhang Z., Li X., Sang S., McClements D.J., Chen L., Long J., Jiao A., Jin Z., Qiu C. (2022). Polyphenols as Plant-Based Nutraceuticals: Health Effects, Encapsulation, Nano-Delivery, and Application. Foods.

[6] Pereira A.G., Echave J., Jorge A.O.S., Nogueira-Marques R., Nur Yuksek E., Barciela P., Perez-Vazquez A., Chamorro F., Maria M.B., Carpena M. (2025). Therapeutic and Preventive Potential of Plant-Derived Antioxidant Nutraceuticals. Foods.

[7] Chhikara N., Kushwaha K., Sharma P., Gat Y., Panghal A. (2019). Bioactive compounds of beetroot and utilization in food processing industry: A critical review. Food Chem..

[8] Thiruvengadam M., Chung I.M., Samynathan R., Chandar S.R.H., Venkidasamy B., Sarkar T., Rebezov M., Gorelik O., Shariati M.A., Simal-Gandara J. (2024). A comprehensive review of beetroot (*Beta vulgaris* L.) bioactive components in the food and pharmaceutical industries. Crit. Rev. Food Sci. Nutr..

[9] Milton-Laskibar I., Alfredo Martínez J., Portillo M.P. (2021). Current Knowledge on Beetroot Bioactive Compounds: Role of Nitrate and Betalains in Health and Disease. Foods.

[10] Clifford T., Howatson G., West D.J., Stevenson E.J. (2015). The Potential Benefits of Red Beetroot Supplementation in Health and Disease. Nutrients.

[11] Baião D.D.S., da Silva D.V.T., Paschoalin V.M.F. (2021). A Narrative Review on Dietary Strategies to Provide Nitric Oxide as a Non-Drug Cardiovascular Disease Therapy: Beetroot Formulations—A Smart Nutritional Intervention. Foods.

[12] Martinez R.M., Melo C.P.B., Pinto I.C., Mendes-Pierotti S., Vignoli J.A., Verri W.A., Casagrande R. (2024). Betalains: A Narrative Review on Pharmacological Mechanisms Supporting the Nutraceutical Potential Towards Health Benefits. Foods.

[13] Nowacka M., Tappi S., Wiktor A., Rybak K., Miszczykowska A., Czyzewski J., Drozdzal K., Witrowa-Rajchert D., Tylewicz U. (2019). The Impact of Pulsed Electric Field on the Extraction of Bioactive Compounds from Beetroot. Foods.

[14] Ersoyak N., Acoglu Celik B., Koc Alibasoglu E., Bekar E., Unal T.T., Yetan E., Kamiloglu S., Akpinar Bayizit A., Copur O.U., Aral Baskaya I. (2025). Production of Red Beetroot (*Beta vulgaris* L.) Pestil as an Alternative Healthy Snack: Effects of Traditional, Thermosonication, and Microwave Pretreatments on Physicochemical, Sensorial, Nutritional, and Chemometric Profiles. Foods.

[15] Zannou O., Oussou K.F., Chabi I.B., Odouaro O.B.O., Deli M.G.E.P., Goksen G., Vahid A.M., Kayodé A.P.P., Kelebek H., Selli S. (2024). A comprehensive review of recent development in extraction and encapsulation techniques of betalains. Crit. Rev. Food Sci. Nutr..

[16] Otálora M.C., Wilches-Torres A., Gómez Castaño J.A. (2023). Microencapsulation of Betaxanthin Pigments from Pitahaya (*Hylocereus megalanthus*) By-Products: Characterization, Food Application, Stability, and In Vitro Gastrointestinal Digestion. Foods.

[17] Sadowska-Bartosz I., Bartosz G. (2021). Biological Properties and Applications of Betalains. Molecules.

[18] Slatnar A., Stampar F., Veberic R., Jakopic J. (2015). HPLC-MSn Identification of Betalain Profile of Different Beetroot (*Beta vulgaris* L. ssp. vulgaris) Parts and Cultivars. J. Food Sci..

[19] Mirmiran P., Houshialsadat Z., Gaeini Z., Bahadoran Z., Azizi F. (2020). Functional properties of beetroot (*Beta vulgaris*) in management of cardio-metabolic diseases. Nutr. Metab..

[20] Jakubczyk K., Melkis K., Janda-Milczarek K., Skonieczna-Żydecka K. (2023). Phenolic Compounds and Antioxidant Properties of Fermented Beetroot Juices Enriched with Different Additives. Foods.

[21] Verma S., Xi Y., Ahsin M., Evans T., van Vliet S. (2026). Conventional, organic, and regenerative organic farming differentially shape nutritional and phytochemical profile in beetroot (*Beta vulgaris*) and its commercial powder products. Front. Nutr..

[22] Sawicki T., Bączek N., Wiczkowski W. (2016). Betalain profile, content and antioxidant capacity of red beetroot dependent on the genotype and root part. J. Funct. Foods.

[23] Punia Bangar S., Singh A., Chaudhary V., Sharma N., Lorenzo J.M. (2023). Beetroot as a novel ingredient for its versatile food applications. Crit. Rev. Food Sci. Nutr..

[24] Yasaminshirazi K., Hartung J., Fleck M., Graeff-Hönninger S. (2021). Impact of cold storage on bioactive compounds and their stability of 36 organically grown beetroot genotypes. Foods.

[25] Wruss J., Waldenberger G., Huemer S., Uygun P., Lanzerstorfer P., Müller U., Höglinger O., Weghuber J. (2015). Compositional characteristics of commercial beetroot products and beetroot juice prepared from seven beetroot varieties grown in Upper Austria. J. Food Compos. Anal..

[26] Carrillo C., Wilches-Pérez D., Hallmann E., Kazimierczak R., Rembiałkowska E. (2019). Organic versus conventional beetroot. Bioactive compounds and antioxidant properties. LWT.

[27] Vasconcellos J., Conte-Junior C., Silva D., Pierucci A.P., Paschoalin V., Alvares T.S. (2016). Comparison of total antioxidant potential, and total phenolic, nitrate, sugar, and organic acid contents in beetroot juice, chips, powder, and cooked beetroot. Food Sci. Biotechnol..

[28] Almeida D., Petropoulos S.A., da Silveira T.F.F., Pires T.C.S.P., Ferreira I.C.F.R., Fernandes Â., Barros L. (2025). Exploring the Biochemical Profile of *Beta vulgaris* L.: A Comparative Study of Beetroots and Swiss Chard. Plants.

[29] Apte M., Nadavade N., Sheikh S.S. (2024). A review on nitrates’ health benefits and disease prevention. Nitric Oxide.

[30] Mella C., Rojas N., Calderon-Bravo H., Muñoz L.A. (2024). Evaluating Biocompounds in Discarded Beetroot (*Beta vulgaris*) Leaves and Stems for Sustainable Food Processing Solutions. Foods.

[31] Lim T.K. (2014). Edible Medicinal and Non Medicinal Plants. Modified Stems, Roots, Bulbs.

[32] Iglesias R., Citores L., Di Maro A., Ferreras J.M. (2014). Biological activities of the antiviral protein BE27 from sugar beet (*Beta vulgaris* L.). Planta.

[33] de Oliveira S.P.A., do Nascimento H.M.A., Sampaio K.B., de Souza E.L. (2021). A review on bioactive compounds of beet (*Beta vulgaris* L. subsp. vulgaris) with special emphasis on their beneficial effects on gut microbiota and gastrointestinal health. Crit. Rev. Food Sci. Nutr..

[34] Coimbra P.P.S., Teixeira Ade C., Trindade M.E.F., Brito G.O., Antonio Ada S., Souza L., Silva-e-Silva ACAGda Pereira H.M.G., Veiga-Junior VFda Felzenszwalb I., Teodoro A.J., Araujo-Lima C.F. (2025). Beetroot peel flour: Characterization, betalains profile, in silico ADMET properties and in vitro biological activity. Food Chem..

[35] Hadipour E., Taleghani A., Tayarani-Najaran N., Tayarani-Najaran Z. (2020). Biological effects of red beetroot and betalains: A review. Phytother. Res..

[36] Mitrevski J., Pantelić N., Dodevska M.S., Kojić J.S., Vulić J.J., Zlatanović S., Gorjanović S., Laličić-Petronijević J., Marjanović S., Antić V.V. (2023). Effect of Beetroot Powder Incorporation on Functional Properties and Shelf Life of Biscuits. Foods.

[37] Buțerchi I., Ciurlă L., Enache I.M., Patraș A., Teliban G.C., Irimia L.M. (2025). Valorisation of Beetroot Peel for the Development of Nutrient-Enriched Dehydrated Apple Snacks. Foods.

[38] Josa E., Barril G., Ruperto M. (2024). Potential Effects of Bioactive Compounds of Plant-Based Foods and Medicinal Plants in Chronic Kidney Disease and Dialysis: A Systematic Review. Nutrients.

[39] Barbu V., Cotarlet M., Bolea C.A., Cantaragiu A., Andronoiu D.G., Bahrim G.E., Enachi E. (2020). Three Types of Beetroot Products Enriched with Lactic Acid Bacteria. Foods.

[40] Guamán-Balcázar Mdel C., Montero M., Celi A., Montes A., Carrera C., Pereyra C., Meneses M.Á. (2024). Encapsulation of Phenolic Compounds Extracted from Beet By-Products: Analysis of Physical and Chemical Properties. Foods.

[41] Calva-Estrada S.J., Jiménez-Fernández M., Lugo-Cervantes E. (2022). Betalains and their applications in food: The current state of processing, stability and future opportunities in the industry. Food Chem. Mol. Sci..

[42] Herbach K.M., Stintzing F.C., Carle R. (2006). Betalain stability and degradation—Structural and chromatic aspects. J. Food Sci..

[43] Corleto K.A., Singh J., Jayaprakasha G.K., Patil B.S. (2018). Storage Stability of Dietary Nitrate and Phenolic Compounds in Beetroot (*Beta vulgaris*) and Arugula (*Eruca sativa*) Juices. J. Food Sci..

[44] Bescos R., Rollason M.L., Davies T.S., Casas-Agustench P. (2023). Content of nitrate and nitrite in commercial and self-made beetroot juices and the effect of storage temperature. Food Sci. Nutr..

[45] Guldiken B., Toydemir G., Nur Memis K., Okur S., Boyacioglu D., Capanoglu E. (2016). Home-Processed Red Beetroot (*Beta vulgaris* L.) Products: Changes in Antioxidant Properties and Bioaccessibility. Int. J. Mol. Sci..

[46] Grace M.H., Hoskin R.T., Alghamdi M., Lila M.A., Chalova V.I. (2025). Betalain–Chickpea Protein Particles Produced by Freeze Drying and Spray Drying: Physicochemical Aspects, Storage Stability, and In Vitro Digestion. Foods.

[47] Martins N., Roriz C.L., Morales P., Barros L., Ferreira I.C.F.R. (2017). Coloring attributes of betalains: A key emphasis on stability and future applications. Food Funct..

[48] Fu Y., Shi J., Xie S.Y., Zhang T.Y., Soladoye O.P., Aluko R.E. (2020). Red Beetroot Betalains: Perspectives on Extraction, Processing, and Potential Health Benefits. J. Agric. Food Chem..

[49] Nistor O.V., Seremet (Ceclu) L., Andronoiu D.G., Rudi L., Botez E. (2017). Influence of different drying methods on the physicochemical properties of red beetroot (*Beta vulgaris* L. var. Cylindra). Food Chem..

[50] Seremet (Ceclu) L., Nistor O.V., Andronoiu D.G., Mocanu G.D., Barbu V.V., Maidan A., Rudi L., Botez E. (2020). Development of several hybrid drying methods used to obtain red beetroot powder. Food Chem..

[51] Kumari S., Goyal A., Sönmez Gürer E., Yapar A., Garg E., Sood M., Sindhu M., Heinämäki J., Kumari S., Goyal A. (2022). Bioactive Loaded Novel Nano-Formulations for Targeted Drug Delivery and Their Therapeutic Potential. Pharmaceutics.

[52] Rahman H.S., Othman H.H., Hammadi N.I., Yeap S.K., Amin K.M., Samad N.A., Alitheen N.B. (2020). Novel Drug Delivery Systems for Loading of Natural Plant Extracts and Their Biomedical Applications. Int. J. Nanomed..

[53] Desseva I., Stoyanova M., Petkova N., Mihaylova D. (2020). Red beetroot juice phytochemicals bioaccessibility: An in vitro approach. Pol. J. Food Nutr. Sci..

[54] Trych U., Buniowska-Olejnik M., Marszałek K. (2022). Bioaccessibility of Betalains in Beetroot (*Beta vulgaris* L.) Juice under Different High-Pressure Techniques. Molecules.

[55] da Silva D.V.T., dos Santos Baião D., de Oliveira Silva F., Alves G., Perrone D., Del Aguila E.M., Flosi Paschoalin V.M. (2019). Betanin, a Natural Food Additive: Stability, Bioavailability, Antioxidant and Preservative Ability Assessments. Molecules.

[56] Igual M., Fernandes Â., Dias M.I., Pinela J., García-Segovia P., Martínez-Monzó J., Barros L. (2023). The In Vitro Simulated Gastrointestinal Digestion Affects the Bioaccessibility and Bioactivity of *Beta vulgaris* Constituents. Foods.

[57] Dalmau M.E., Llabrés P.J., Eim V.S., Rosselló C., Simal S. (2019). Influence of freezing on the bioaccessibility of beetroot (*Beta vulgaris*) bioactive compounds during in vitro gastric digestion. J. Sci. Food Agric..

[58] Shahidi F., Pan Y. (2022). Influence of food matrix and food processing on the chemical interaction and bioaccessibility of dietary phytochemicals: A review. Crit. Rev. Food Sci. Nutr..

[59] Hu Y., Lin Q., Zhao H., Li X., Sang S., McClements D.J., Long J., Jin Z., Wang J., Qiu C. (2023). Bioaccessibility and bioavailability of phytochemicals: Influencing factors, improvements, and evaluations. Food Hydrocoll..

[60] Clifford T., Constantinou C.M., Keane K.M., West D.J., Howatson G., Stevenson E.J. (2016). The plasma bioavailability of nitrate and betanin from *Beta vulgaris* rubra in humans. Eur. J. Nutr..

[61] Fadeel D.A., Fadel M., El-Kholy A.I., El-Rashedy A.A., Mohsen E., Ezzat M.I., Issa M.Y. (2024). Nano-Liposomal Beetroot Phyto-Pigment in Photodynamic Therapy as a Prospective Green Approach for Cancer Management: In Vitro Evaluation and Molecular Dynamic Simulation. Pharmaceutics.

[62] Sheir M.A., Ramadan M.M., El-Messery T.M., Mohamed E.N. (2025). Enhancing iron bioavailability and bioactive stability in sweet-sour beetroot sauce through liposomal vitamin C. Food Chem. Adv..

[63] Kaushik R., Sharma V., Kumar K., Sharma R., Gaur P.K. (2026). Design and Development of Betacyanin-Encapsulated Solid Lipid Nanoparticles for Antidiabetic Therapy. J. Appl. Pharm. Sci. Res..

[64] Kassem A.A., Abd El-Alim S.H., Salman A.M., Mohammed M.A., Hassan N.S., El-Gengaihi S.E. (2020). Improved hepatoprotective activity of *Beta vulgaris* L. leaf extract loaded self-nanoemulsifying drug delivery system (SNEDDS): In vitro and in vivo evaluation. Drug Dev. Ind. Pharm..

[65] Tran Q.H., Chu H.K.T., Nguyen P.T., Nguyen V.M., Nguyen Q.T., Tran C.D., Nguyen T.D. (2023). Double Nanoemulsion Loading Betalains Extract of Beetroot (*Beta vulgaris* L.): Ultrasound-Assisted Synthesis, Storage Stability, and Antioxidant Activity. ACS Food Sci. Technol..

[66] Silva Nieto R., Samaniego López C., Moretton M.A., Lizarraga L., Chiappetta D.A., Alaimo A., Pérez O.E. (2023). Chitosan-Based Nanogels Designed for Betanin-Rich Beetroot Extract Transport: Physicochemical and Biological Aspects. Polymers.

[67] El-ghannam G., Moawad M., Abo-Elfadl M.T., Elfeky S.A. (2024). Beetroot extract@chitosan nanocomposite as a promising approach towards cancer therapy. Int. J. Biol. Macromol..

[68] Ghoorchi A., Arianfar A., Hakimzadeh V., Naji-Tabasi S. (2025). Nano-Encapsulation of Red Beetroot Extract Based on Betanin With Gelatin and Cress Seed Gum by Coacervation Technique. Food Sci. Nutr..

[69] Tavana F., Hematian Sourki A., Golmakani M.T. (2024). Enhancing the accuracy, speed, and efficiency of kafirin-PEO electrospun bio-nanocomposite pH indicators with red beetroot extract using image processing. Food Sci. Nutr..

[70] Jenikha J.S.A., Rajiv S. (2026). Electrospun zein nanofibers containing *Beta vulgaris* betalains and Bromocresol purple dye as pH-sensitive colorants for monitoring fruit spoilage. Color. Technol..

[71] Khattab T.A., Ahmed H.M., Zhou Y., Ding X., Abdelrahman M.S., Hassabo A.G. (2025). Development of Betalain-immobilized polylactic acid nanofibers as a green and sustainable sensor for toxic ammonia. Int. J. Biol. Macromol..

[72] Viegas C., Patrício A.B., Prata J.M., Nadhman A., Chintamaneni P.K., Fonte P. (2023). Solid Lipid Nanoparticles vs. Nanostructured Lipid Carriers: A Comparative Review. Pharmaceutics.

[73] Haddadzadegan S., Dorkoosh F., Bernkop-Schnürch A. (2022). Oral delivery of therapeutic peptides and proteins: Technology landscape of lipid-based nanocarriers. Adv. Drug Deliv. Rev..

[74] Hsu C.Y., Wang P.W., Alalaiwe A., Lin Z.C., Fang J.Y. (2019). Use of Lipid Nanocarriers to Improve Oral Delivery of Vitamins. Nutrients.

[75] Alharbi W.S., Almughem F.A., Almehmady A.M., Jarallah S.J., Alsharif W.K., Alzahrani N.M., Alshehri A.A. (2021). Phytosomes as an Emerging Nanotechnology Platform for the Topical Delivery of Bioactive Phytochemicals. Pharmaceutics.

[76] Harwansh R.K., Deshmukh R., Shukla V.P., Khunt D., Prajapati B.G., Rashid S., Ali N., Elossaily G.M., Suryawanshi V.K., Kumar A. (2024). Recent Advancements in Gallic Acid-Based Drug Delivery: Applications, Clinical Trials, and Future Directions. Pharmaceutics.

[77] Martínez-Rodríguez P., Guerrero-Rubio M.A., Hernández-García S., Henarejos-Escudero P., García-Carmona F., Gandía-Herrero F. (2023). Characterization of betalain-loaded liposomes and its bioactive potential in vivo after ingestion. Food Chem..

[78] Choi S.J., McClements D.J. (2020). Nanoemulsions as delivery systems for lipophilic nutraceuticals: Strategies for improving their formulation, stability, functionality and bioavailability. Food Sci. Biotechnol..

[79] Jacob S., Rao R., Gorain B., Boddu S.H.S., Nair A.B. (2025). Solid Lipid Nanoparticles and Nanostructured Lipid Carriers for Anticancer Phytochemical Delivery: Advances, Challenges, and Future Prospects. Pharmaceutics.

[80] Mall J., Naseem N., Haider M.F., Rahman M.A., Khan S., Siddiqui S.N. (2025). Nanostructured lipid carriers as a drug delivery system: A comprehensive review with therapeutic applications. Intell. Pharm..

[81] Soufiani S.P., Yousefi S., Honarvar M., Weisany W., Asadi G. (2025). Bioactive chitosan packaging films with encapsulated beet leaf extract for preserving chicken fillets. Food Chem. X.

[82] Singh D.P., Gopinath P. (2023). Tragacanth gum-based nano-nutraceuticals synthesis by encapsulation of beetroot juice and Ocimum basilicum leaves for micronutrient deficient population. Int. J. Biol. Macromol..

[83] Akbari-Alavijeh S., Shaddel R., Jafari S.M. (2020). Encapsulation of food bioactives and nutraceuticals by various chitosan-based nanocarriers. Food Hydrocoll..

[84] de Barros Alexandre J., Barroso T.L.C.T., da Silva L.C., Castelo R.M., Freire G.A., Nascimento A.B., Cheng H.N., Biswas A., Bruno L.M., Furtado R.F. (2024). Encapsulation of beetroot extract (*Beta vulgaris* L.) obtained by internal and external ionic gelation: A comparative study. Food Sci. Technol..

[85] Chaux-Gutiérrez A.M., Pérez-Monterroza E.J., Granda-Restrepo D.M., Mauro M.A. (2020). Cryogels from albumin and low methoxyl amidated pectin as a matrix for betalain encapsulation. J. Food Process. Preserv..

[86] Chaux-Gutiérrez A.M., Pérez-Monterroza E.J., Granda-Restrepo D.M., Mauro M.A. (2020). Effect of temperature and relative humidity on the stability of betalains encapsulated in cryogels from protein and polysaccharide. J. Food Sci. Technol..

[87] Prakasha R., Vinay G.M., Pathiam S., Hridyesh P. (2025). Nanoemulsions as Carriers of Bioactive Compounds in Functional Foods: Preparation and Application. Eur. J. Nutr. Food Saf..

[88] Rezagholizade-shirvan A., Soltani M., Shokri S., Radfar R., Arab M., Shamloo E. (2024). Bioactive compound encapsulation: Characteristics, applications in food systems, and implications for human health. Food Chem. X.

[89] Waqar M., Vasanthkumar S.S., Ullah Q., Awlqadr F.H., Arshad M.T., Panpipat W., Chaijan M., Kirkusawi S.H.M., Laryea E. (2026). Nanoencapsulation in functional foods: Improving delivery, stability, and sustainability of bioactive compounds. Int. J. Food Sci. Technol..

[90] Gul K., Choudhury D.B., Hosseinpur A., Roques-Carmes T. (2026). Recent developments in encapsulation technologies for stabilizing natural pigments in food systems: Mechanisms, selection strategies, and industrial feasibility. Food Biosci..

[91] Guan Y., Ning Y., Xu Z., Zhou C., Zhao Z. (2024). Chondroitin sulfate and chitosan-coated liposomes as a novel delivery system for betanin: Preparation, characterization and in vitro digestion behavior. Int. J. Biol. Macromol..

[92] Trindade L.R.d., Baião D.d.S., da Silva D.V.T., Almeida C.C., Pauli F.P., Ferreira V.F., Conte-Junior C.A., Paschoalin V.M.F. (2023). Microencapsulated and Ready-to-Eat Beetroot Soup: A Stable and Attractive Formulation Enriched in Nitrate, Betalains and Minerals. Foods.

[93] Čakarević J., Torbica A., Belović M., Tomić J., Sedlar T., Popović L. (2021). Pumpkin oil cake protein as a new carrier for encapsulation incorporated in food matrix: Effect of processing, storage and in vitro digestion on bioactivity. Int. J. Food Sci. Technol..

[94] Qazi H.J., Ye A., Acevedo-Fani A., Singh H. (2025). Delivery of encapsulated bioactive compounds within food matrices to the digestive tract: Recent trends and future perspectives. Crit. Rev. Food Sci. Nutr..

[95] Meng R., Wu Z., Xie Q.T., Cheng J.S., Zhang B. (2021). Preparation and characterization of zein/carboxymethyl dextrin nanoparticles to encapsulate curcumin: Physicochemical stability, antioxidant activity and controlled release properties. Food Chem..

[96] McClements D.J. (2021). Advances in edible nanoemulsions: Digestion, bioavailability, and potential toxicity. Prog. Lipid Res..

[97] Paredes-Toledo J., Herrera J., Morales J., Robert P., Gómez-Estaca J., Giménez B. (2025). Pickering Double Emulsions Stabilized with Chitin Nanocrystals and Myristic Acid-Functionalized Silica Nanoparticles for Curcumin and Chlorogenic Acid Co-Delivery. Pharmaceutics.

[98] Amjadi S., Ghorbani M., Hamishehkar H., Roufegarinejad L. (2018). Improvement in the stability of betanin by liposomal nanocarriers: Its application in gummy candy as a food model. Food Chem..

[99] Amjadi S., Mesgari Abbasi M., Shokouhi B., Ghorbani M., Hamishehkar H. (2019). Enhancement of therapeutic efficacy of betanin for diabetes treatment by liposomal nanocarriers. J. Funct. Foods.

[100] Liu S., Lian J., Xu Z., Ning Y., Shi M., Zhao Z., Zhang Z. (2022). Chitosan-coated nanoliposomes for efficient delivery of betanin with enhanced stability and bioavailability. Food Hydrocoll..

